# Inefficient antiviral response in reconstituted small-airway epithelium from chronic obstructive pulmonary disease patients following human parainfluenza virus type 3 infection

**DOI:** 10.1186/s12985-024-02353-7

**Published:** 2024-04-02

**Authors:** Louise Bondeelle, Maud Salmona, Véronique Houdouin, Elise Diaz, Jacques Dutrieux, Séverine Mercier-Delarue, Samuel Constant, Song Huang, Anne Bergeron, Jérôme LeGoff

**Affiliations:** 1 Department of Microbiology and Molecular Medicine, University of Geneva, Geneva, Switzerland; 2 Virology Department, AP-HP, Hôpital Saint-Louis, 1 Avenue Claude Vellefaux, Paris, F-75010 France; 3 Service de Pneumologie, APHP, Hôpital Robert-Debré, Paris, F-75010 France; 4 Université Paris Cité, Inserm U976, INSIGHT Team, Paris, F-75010 France; 5 Université Paris Cité, Institut Cochin, INSERM, U1016, CNRS, UMR8104, Paris, F-75014 France; 6 Epithelix Sarl, Geneva, 1228 Switzerland; 7 Pneumology Department, Geneva University Hospitals, Geneva, Switzerland

**Keywords:** Transcriptome, Respiratory virus, Pathogenesis, Airway epithelium

## Abstract

Chronic obstructive pulmonary disease (COPD) affects over 250 million individuals globally and stands as the third leading cause of mortality. Respiratory viral infections serve as the primary drivers of acute exacerbations, hastening the decline in lung function and worsening the prognosis. Notably, Human Parainfluenza Virus type 3 (HPIV-3) is responsible for COPD exacerbations with a frequency comparable to that of Respiratory Syncytial Virus and Influenza viruses. However, the impact of HPIV-3 on respiratory epithelium within the context of COPD remains uncharacterized.

In this study, we employed in vitro reconstitution of lower airway epithelia from lung tissues sourced from healthy donors (*n* = 4) and COPD patients (*n* = 5), maintained under air–liquid interface conditions. Through a next-generation sequencing-based transcriptome analysis, we compared the cellular response to HPIV-3 infection.

Prior to infection, COPD respiratory epithelia exhibited a pro-inflammatory profile, notably enriched in canonical pathways linked to antiviral response, B cell signaling, IL-17 signaling, and epithelial-mesenchymal transition, in contrast to non-COPD epithelia. Intriguingly, post HPIV-3 infection, only non-COPD epithelia exhibited significant enrichment in interferon signaling, pattern recognition receptors of viruses and bacteria, and other pathways involved in antiviral responses. This deficiency could potentially hinder immune cell recruitment essential for controlling viral infections, thus fostering prolonged viral presence and persistent inflammation.

**Supplementary Information:**

The online version contains supplementary material available at 10.1186/s12985-024-02353-7.

## Introduction

Chronic obstructive pulmonary disease (COPD) impacts over 250 million individuals globally and ranks as the third leading cause of mortality. Acute exacerbations in COPD can precipitate a hastened decline in lung function and worsen prognoses. Among the culprits of exacerbations, respiratory viral infections play a prominent role [1]. Among respiratory viruses, influenza, rhinovirus and respiratory syncytial virus (RSV) are frequently involved. Though less scrutinized, compelling epidemiological evidence positions Human Parainfluenza Virus type 3 (HPIV-3) on par with RSV in exacerbating COPD [2]. Recent prospective research [3] underscored that HPIV, Influenza B virus, and RSV B exhibited the highest odds ratios for acute exacerbation events. Nonetheless, the comprehensive pathogenicity of HPIV-3 warrants further exploration [4, 5]. The respiratory epithelia, encompassing upper and lower tracts, serve as primary targets for respiratory viruses and constitute the first line of defense. They orchestrate immune responses via the production of interferons, cytokines, and chemokines, thus directing systemic and adaptive defenses [6]. This study aims to decode the epithelial cell response to HPIV-3 infection through transcriptome analysis in air-liquid interface (ALI)-differentiated respiratory epithelium and to compare results obtained in reconstituted epithelium from COPD and non-COPD subjects.

## Materials and methods

### Human airway epithelia (HAE)

We used human lower airway epithelia reconstituted in vitro (SmallAir™, Epithelix, Geneva, Switzerland) from lung biopsies from healthy donors (*n* = 4) and patients with chronic obstructive pulmonary disease (*n* = 5). Patient characteristics and cause of death are reported in Table 1.

**Table 1:** Characteristics of COPD and non-COPD individuals

	Gender	Age (years)	Smoking history (PY)	Cause of death	Medical history
#2	M	60	45	Cerebrovascular /stroke	COPD
#5	M	58	25	Cerebrovascular /stroke	COPD
#7	M	75	200	Cerebrovascular /stroke	COPD
#8	F	61	90	Anoxia (COPD exacerbation)	COPD
#9	M	53	20	Anoxia (secondary to cardiovascular disease)	COPD
#1	M	19	0	Head trauma	None
#3	F	56	0	Cerebrovascular /stroke	None
#6	F	71	1	Cerebrovascular /stroke	None
#10	F	61	0	Cerebrovascular /stroke	None

SmallAir™ tissues were cultured in an air-liquid interface (ALI) system at 37 °C under a 5% of CO2 atmosphere following manufacturer’s recommendations. The basolateral medium was changed every 2 days until infection.

### Virus

HPIV-3 (MK9 strain, Public Health England – Culture Collection), was amplified in A549 cells. For viral stock, cells were infected with a TCID50 of 1,72 × 10^10^ in DMEM with 2% SVF for 1 h at 37 °C. Fresh medium with 2% SVF was then added. The inoculum was removed 24 h post infection (hpi). The infectious supernatant was collected 72 hpi, aliquoted, and frozen at -80 °C before titration. All experimental work was performed in a biosafety level 2 laboratory.

### Cell cultures infection

The apical surface of SmallAir™ was inoculated with 100 μL of 1 × 10^8^ TCID50 of HPIV-3 (multiplicity of infection (MOI) = 10) during 3 h at 37 °C under 5% of CO2. The inoculum was then removed and apical PBS washes were performed. To quantify HPIV-3 replication, HPIV-3 RNA was quantified at the apical surface at different times post-inoculation by quantitative real-time PCR. Apical PBS washes were collected before infection, 3 hpi, 24 hpi, 48 hpi and 96 hpi.

### HPIV-3 RNA quantification

Viral RNA was extracted from 50μl of SmallAir™ apical washes and eluted in 50μl using NucliSENS easyMAG (biomérieux). HPIV-3 RNA was reverse-transcribed with SuperScript VILO cDNA Synthesis Kit and then amplified using TaqMan Universal PCR Master Mix, and a specific set of primers and probe targeting HPIV-3 matrix gene (forward primer 5’-ATTTTATGCTCCTATCTAGTGGAAGACA-3’, reverse primer 5’-TGCTGTTCGATGCCAACAA-3’, probe 5’-FAM-TTGCTCTTGCTCCTCA-MGB-3’) on an ABI 7500 thermocycler (ThermoFisher). A standard curve was established with the strain MK-9 HPIV-3.

### RNA sequencing

After 48 hpi, RNA isolation was carried out from 3 apical inserts. The cells were washed twice with media and PBS and then lysed in 40 μl of RNA Protect Cell Reagent (Qiagen, Hilden, Germany) and 200 μl of media. The suspension was centrifuged for 5 min at 5,000 g in order to obtain a pellet and the supernatant was removed. The pellet was suspended in 300 μl of RNA Protect Cell Reagent and frozen at -80 °C until preparation of cDNA libraries. cDNA libraries were prepared from 0.5 μg of total RNA using the TruSeq RNA Library Prep Kit v2 (Illumina, SanDiego, California) according to manufacturer’s instructions. Prior to sequencing, libraries were quantified with kappa library quantification kit (Roche, Bâle, Swiss) and quality was assessed with High Sensitivity RNA ScreenTape on a 2200 TapeStation system (Agilent Technologies). All libraries were pooled and sequencing was performed on Nextseq 550 (Illumina) with a 75 × 2 cartridge.

### Bioinformatics analysis

Conversion and demultiplexing of reads were performed using bcl2fastq. FastQC was used for quality controls of the raw data. Reads were cleaned using the Trimmomatic software with a minimum quality threshold of Q30 [7]. Cleaned reads were aligned and counted to the annotated Homo sapiens genome (GRCh38.p11) using STAR software [8]. Principal component analysis and identification of differentially expressed genes were performed using the R package DESeq2 [9]. Genes with an absolute fold change greater than two and an adjusted *p*-value less than 0.05 (FDR < 5%) were considered to be differentially expressed. Ontological analyses were performed with IPA software (Qiagen). In addition to determining the enriched biological pathways for each comparison (Fisher’s exact test, *p*-value < 0.05), for each enriched biological pathway, the software calculate a Z score. The Z score determines whether the changes in gene expression for the known genes of each biological pathway are consistent with what is expected from the literature (Z score p).

## Results

Apical HPIV-3 loads were evaluated at 0, 24, 48, and 96 hpi. Notably, no statistically significant differences were observed when comparing COPD and non-COPD epithelia (Figure S1). Microscopic examination (not shown) revealed a greater production of mucus both before and after infection in COPD epithelia compared to non-COPD epithelia consistent with clinical observations [10].

RNA sequencing provided a median count of 37,359,727 reads per sample, with 85 to 95% of reads successfully mapped to the human genome (Table S1). The sequencing encompassed more than 20,000 human genes for each condition.

In the basal state, a comparative analysis of transcriptomic profiles was performed between COPD and non-COPD non infected epithelia. Distinct differences emerged in COPD epithelia, characterized by specific enrichments in canonical pathways linked to antiviral response, B cell signaling, IL-17 signaling, and the regulation of epithelial-mesenchymal transition by growth factors, in contrast to non-COPD epithelia (Table 2).

To assess the transcriptional alterations elicited by HPIV-3 infection in both non-COPD and COPD epithelia, we identified differentially expressed (DE) genes (fold change > |2|, FDR < 5%) between infected and uninfected epithelia in both conditions. The most significant variations in expression were observed between infected COPD and uninfected COPD epithelia, revealing 4017 DE genes (with 2186 being downregulated and 1831 upregulated). Meanwhile, between non-COPD infected and uninfected epithelia, we identified a sum of 553 DE genes (comprising 65 downregulated and 488 upregulated).

**Table 2:** Enriched canonical pathways between SmallAir epithelia obtained from healthy donors and from patients with COPD. The Z score determines whether the changes in gene expression for the known genes of each biological pathway are consistent with what is expected from the literature (Z score positive) or if the changes are inversely correlated with the literature (Z score negative)

Canonical Pathways	z-score
Role of Hypercytokinemia/hyperchemokinemia in the Pathogenesis of Influenza	3.16
Systemic Lupus Erythematosus In B Cell Signaling Pathway	2.83
Interferon Signaling	2.53
IL-17 Signaling	2.45
Role of MAPK Signaling in Inhibiting the Pathogenesis of Influenza	2.24
Regulation Of The Epithelial Mesenchymal Transition By Growth Factors Pathway	2.00
Estrogen Receptor Signaling	2.00
HIF1α Signaling	2.00
Activation of IRF by Cytosolic Pattern Recognition Receptors	1.34
Tumor Microenvironment Pathway	1.34
Hepatic Fibrosis Signaling Pathway	1.34
Acute Phase Response Signaling	1.00
MSP-RON Signaling In Macrophages Pathway	1.00
Leukocyte Extravasation Signaling	1.00
Adrenomedullin signaling pathway	1.00
Colorectal Cancer Metastasis Signaling	1.00
Dendritic Cell Maturation	1.00
Neuroinflammation Signaling Pathway	0.82
Coronavirus Pathogenesis Pathway	0.33
Sirtuin Signaling Pathway	-1.34

**Fig. 1:**
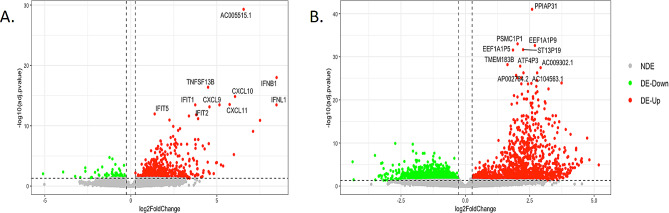
Volcanoplot of upregulated and downregulated DE genes after 48 h post HPIV3 infection in SmallAir epithelia obtained from healthy donors (**A**) and from patients with COPD (**B**)

The Volcanoplot in Fig. 1 illustrates the prominently upregulated and downregulated DE genes in both comparative analyses. Among the top 10 DE genes in non-COPD epithelia, we note the presence of interferons, proteins induced by interferons (such as IFIT, CXCL 9–11), and TNF, collectively suggesting a robust and distinctive antiviral response. In contrast, the infection of COPD epithelia did not yield a discernible upregulation of a specific antiviral response.

**Fig. 2:**
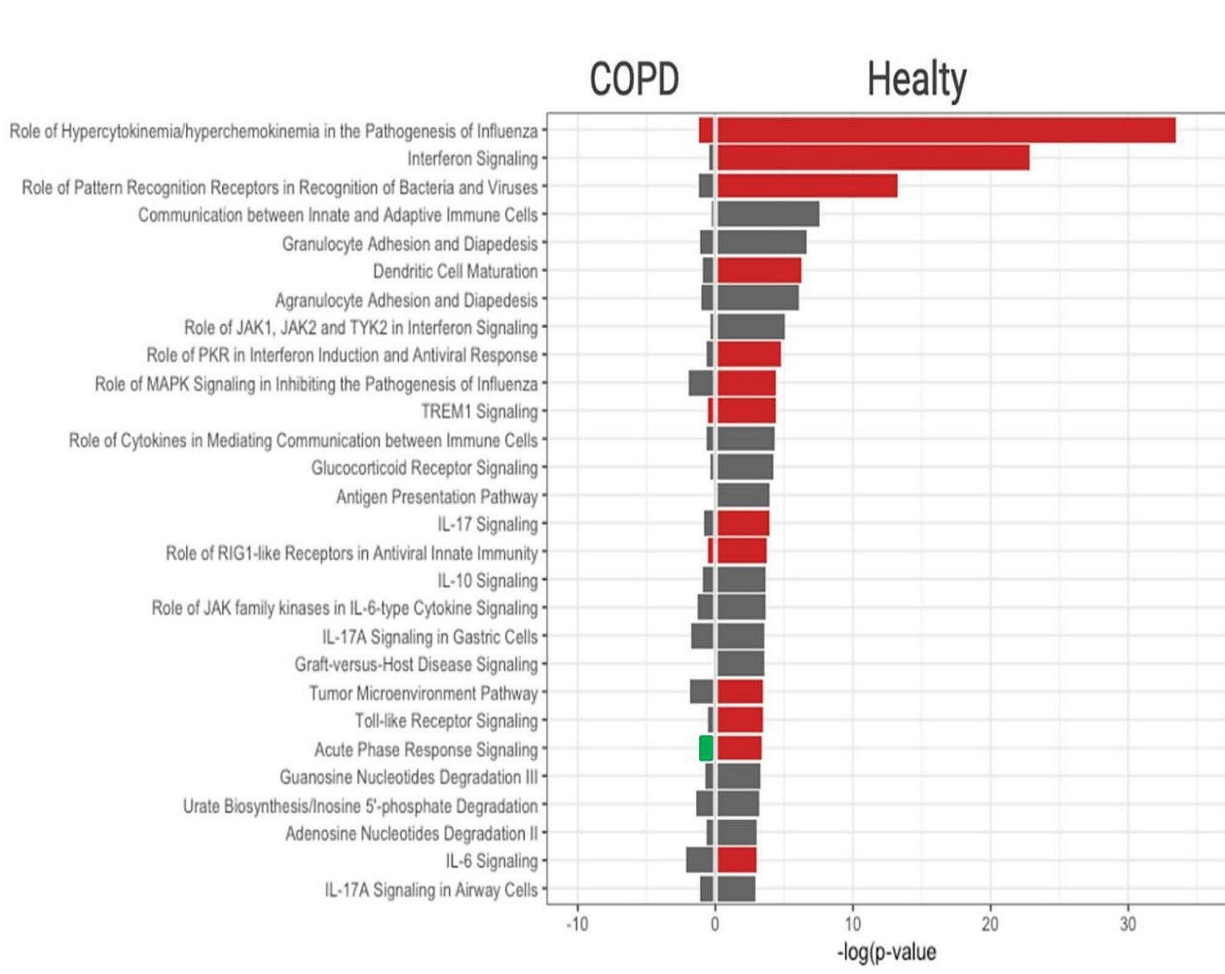
Enriched biological pathways after HPIV-3 infection. Comparison of significantly enriched biological pathways after 48 h post HPIV3 infection in SmallAir epithelia obtained from healthy donors and from patients with COPD. Log_10_ (*p* value) is represented on x axis (threshold = 1.3). Predicted activated pathway (z-score > 1) are in red font. predicted inhibited pathway (z-score<-1) in green and baseline are in grey

We further examined the impact of HPIV-3 on biological cellular pathways. All pathways significantly enriched after infection are listed in the Supplementary Table S2 for both non-COPD and COPD epithelia. Importantly, in non-COPD epithelia, the aftermath of HPIV-3 infection heralded substantial enrichments in interferon signaling, pattern recognition receptors of viruses and bacteria, as well as other pivotal pathways integral to antiviral responses (comprising Dendritic Cell Maturation, TLR Signaling, MAPK Signaling, and the Antigen Presentation Pathway). Conversely, this discernible enrichment was notably absent in COPD epithelia (Fig. 2).

## Discussion

Previous works showed that bronchial epithelium reconstituted from airway tissues of COPD patients cultured in ALI, recapitulated phenotypic epithelial characteristics observed in lung biopsy samples, with a decrease of ciliated cells and an increase of goblet cells [11]. Distinct gene sets distinguishing chronic obstructive pulmonary disease from healthy lung samples in the absence of viral infection were identified in other investigations [12–14]. The present study showed that reconstituted epithelia from COPD and non-COPD donors present also distinguished cellular response to viral infection 48 h after infection. First, prior to infection, COPD airway epithelia displayed an elevated baseline expression of antiviral and epithelial-mesenchymal transition pathways compared to their non-COPD counterparts. Secondly, despite observing a more pronounced divergence in gene expression 48 h after HPIV-3 infection, COPD epithelia exhibited an impairment in mounting a specific antiviral response. This was particularly evident in the Interferon signaling, IL17, IL10, and IL6 signaling pathways, as well as the Dendritic Cell Maturation, TLR signaling, MAPK signaling, and Antigen presentation pathway. This impediment could hinder the recruitment of immune cells, thereby fostering prolonged infection. At basal state in COPD epithelia, activated pathways of IL-17 signaling and epithelial-mesenchymal transition induced by growth factors may play a role in virus-induced lung inflammation, as previously reported with RSV. IL-17 is associated with airway hyperresponsiveness and is implicated in the recruitment of inflammatory cells, contributing to lung remodeling [15]. In chronic inflammation respiratory epithelial models, IL-17 is involved in increased mucus production and cell hyperplasia after RSV infection [16] and may promote the accumulation of neutrophils and worsen the mucosal inflammation [17]. The epithelial-mesenchymal transition has been shown to decrease mucosal antiviral response [18]. Finally, the baseline activation of HIF-1alpha in COPD epithelia known as a negative regulator of CD4 and CD8 T cells [19] may also alter adaptive immune response.

Thus, despite activated interferon signaling, the basal activation of other pathways may, in contrast, counteract the likely antiviral response. This upregulated inflammatory process could contribute to worsened airways obstruction through sustained goblet cell hyperplasia and mucin overproduction following HPIV-3 infection, as seen in other viruses affecting COPD differentiated epithelia [20].

Furthermore, the absence of an inducible antiviral profile after HPIV-3 infection may increase susceptibility or worsen epithelial damage after a subsequent heterologous viral infection [21].

Very few studies have investigated HPIV-3 infection in differentiated pseudostratified respiratory epithelium at the air-liquid interface and only with samples collected from healthy donors [5, 22, 23]. Those studies employed slightly lower inoculum with MOI ranging from 3 to 6 MOI than that used in our study [10]. The results of one study investigating the kinetics of viral replication and cytokine release [23], corroborate our findings. Indeed, HPIV-3 replication peaked at 48 h post-infection at 10^7.9^ TCID50/ml and remained at a steady state for 5 days, and a progressive increase in the production of cytokines started at 48 hpi. Our preliminary findings warrant further functional studies to complement the gene expression profile results.

Our observations might elucidate the propensity of infected COPD epithelia to exacerbate inflammation in response to viral infection, analogous to observations in asthmatic patients with Rhinovirus or Respiratory Syncytial Virus [24]. Interestingly, a heightened baseline antiviral response in COPD was documented in a study involving Rhinovirus infection [25]. In a similar model of differentiated primary epithelial cells obtained from healthy and COPD donors, Chander Veerati et al. observed that COPD did not exhibit a defect in antiviral response to Rhinovirus (Species A, type 1) infection. Rather, a delay of 48 h was observed in differentially expressed genes, particularly within the innate immunity pathway [26]. This underscores diverse patterns of epithelial responses in COPD contingent upon the viral species.

While current therapies effectively mitigate chronic symptoms, their efficacy in preventing COPD exacerbations remains limited. Deciphering the intricate mechanisms through which bronchiolar epithelia respond to respiratory viruses and subsequently trigger chronic inflammation holds significant potential in devising targeted strategies to forestall the alteration of respiratory functions subsequent to viral infections. Air-liquid interface differentiated respiratory epithelia stand as promising models to explore novel therapeutic avenues.

### Electronic supplementary material

Below is the link to the electronic supplementary material.

Supplementary Material 1

Supplementary Material 2

Supplementary Material 3

## Data Availability

No datasets were generated or analysed during the current study.
