# Supplementary figures and images for: Inefficient antiviral response in reconstituted small-airway epithelium from chronic obstructive pulmonary disease patients following human parainfluenza virus type 3 infection
Source: Virol J. 2024 Apr 2;21:78. doi: 10.1186/s12985-024-02353-7 (PMC10988791; doi:10.1186/s12985-024-02353-7)

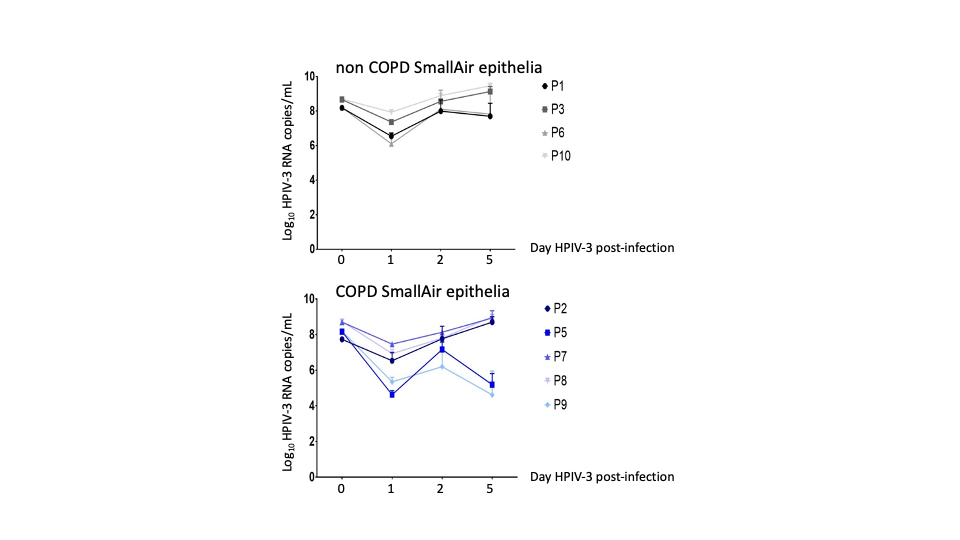

Supplement: Supplementary file 1 — Supplementary Material 1 [file 12985_2024_2353_MOESM1_ESM.png]
